# Microstructural Study of Arc Beads in Aluminum Alloy Wires with an Overcurrent Fault

**DOI:** 10.3390/ma14154133

**Published:** 2021-07-24

**Authors:** Xueyan Xu, Zhijin Yu, Yang Li, Weifeng Wang, Lan Xu

**Affiliations:** 1 Department of Justice Technology, Jilin Justice Officer Academy, Changchun 130062, China; xyxu0108@163.com; 2 College of Safety Science and Engineering, Xi’an University of Science and Technology, Xi’an 710054, China; li_yang@cppu.edu.cn (Y.L.); guyu199669@163.com (W.W.); 15898395352@163.com (L.X.); 3 School of Investigation, Chinese People’s Police University, Langfang 065000, China

**Keywords:** electrical fire, aluminum alloy wires, overcurrent fault, arc beads, microstructure

## Abstract

To clarify the understanding and analysis of arc molten marks in electrical faults of aluminum alloy wires, this paper simulates overcurrent faults of aluminum alloy wires at currents of 128 A–224 A and uses thermogravimetry-differential scanning calorimetry (TG-DSC), optical microscope (OM), scanning electron microscope (SEM) and X-ray energy spectroscopy (EDS) to characterize the effects of current on the microstructure of arc beads. The results show that there are small and large amounts of Al-Si and Al-Fe binary phases in the metallographic structure of the aluminum alloy wires at the rated current, the grains are fine, and there are no significant grain boundaries. After an overcurrent fault occurs in the wires, a high-temperature arc causes the second phase in the aluminum alloy to disappear, a cellular dendritic metallographic structure appears, the grain boundaries become more well-defined, and composition segregation occurs at the grain boundaries. Using Image-Pro-Plus software to quantify the grain characteristics, the average grain size is found to gradually decrease as the current increases. In addition, by comparing and analyzing the characteristics of arc beads in aluminum wires and aluminum alloy wires under the same conditions, alloying elements are found to have a refining effect on the grain boundaries, and there are coarse precipitates at the grain boundaries in the aluminum wire arc beads.

## 1. Introduction

Aluminum alloy wires are widely used in high-voltage transmission lines and power supply mains due to their strong oxidation resistance, low density and cost effectiveness [1,2,3]. In recent years, molten marks from the electrical failure of aluminum alloy wires have been found at fire scenes. Electrical fires can be classified according to their mechanism of initiation, i.e., poor connections [4], short-circuits [5], externally induced ionization of air [6], carbonization of insulation [7], overcurrents [8], hot particles [9], solid-liquid insulation breakdown and other mechanisms [10]. Because the mechanisms of electrical fires and the environments in which they are located differ, the microscopic characteristics of the molten marks remaining after failure also have their own characteristics. Fire investigators determine the fire point by arc mapping [11], then analyze the characteristics of molten marks at the fire point, invert the fire scene fault, and finally determine the cause of the fire.

Researchers at home and abroad have conducted many studies on molten marks around fire points. The National Fire Protection Association (NFPA) 921 [12] describes the characteristics of arc beads from short-circuit and molten marks from wires that caused fires. Erlandsson and Strand [13] used scanning electron microscope (SEM) to observe short-circuit and overcurrent fault arc beads and found many holes in short-circuit arc beads formed in an air environment but no holes in overcurrent fault arc beads. Takaki [14] used a optical microscope (OM) to survey the microstructural characteristics of short-circuit fault arc beads and found that the metallographic structure of short-circuit arc beads is dominated by fine columnar crystals and cellular crystals. Babrauskas V [15] found carbon-containing materials in the “cause” beads through X-ray energy spectroscopy (EDS), but no carbon was found in the “victim” beads. Wu Ying [16,17] used the Auger electron spectroscopy (AES) technology to analyze the influence of the oxygen concentration on the characteristics of short-circuit arc beads, and the results showed that there was a positive linear correlation between the average oxygen content on the surface of short-circuit arc beads and ambient oxygen concentration. Wang Hairong [18] used X-ray diffraction (XRD) to study the phase structure characteristics of short-circuit arc beads. The results showed that the Cu_2_O content of the cubic crystal system was lowest in the short-circuit “cause” beads, the Cu and Cu_2_O diffraction peaks in the short-circuit “victim” beads were broadened, cracking occurred, the Cu_2_O content for the overcurrent fault was approximately 30%, and the Cu_2_O equiaxial crystal diffraction peak intensity was the largest in the fire molten marks. Park [19] used electron backscatter diffraction to distinguish copper molten marks. The globules did not show evident demarcation lines, and the microstructure consisted of globular or dendritic grains; the primary arc beads (PABs) exhibited a strong (001) texture perpendicular to the demarcation lines and comprised large fractions of columnar grains with a small grain aspect ratio (GAR). The microstructure of the secondary arc beads (SABs) was a mixture of elongated and equiaxial grains with a large GAR and no specific development of texture.

At home and abroad, the microstructure of the electrical fault molten marks of copper and aluminum wires has been studied from different angles [20,21,22,23]. However, there are few studies on the electrical fault molten marks of aluminum alloys and other new types of wires. This is not very useful for determining the cause of electrical fire associated with aluminum alloy wires. What are the differences between the electrical fault arc beads of aluminum alloy wires and those of aluminum wires? Do alloying elements affect electric fire molten marks? This is an issue that urgently needs to be solved to support the investigation of evidence and identification of sources of fires. Babrauskas V [24] noted that overcurrent faults have higher fire hazards. In addition, overcurrent is the ultimate manifestation of a variety of electrical faults [25,26]. This article is based on previous research. Through thermogravimetry-differential scanning calorimetry (TG/DSC), OM, SEM and EDS, the microscopic characteristics of arc beads of aluminum alloy wires with an overcurrent fault are explored, and the influence of current on the characteristics of the arc beads is investigated, providing a scientific reference for the analysis of evidence from fires induced by overcurrent faults.

## 2. Materials and Methods

**Figure 1:**
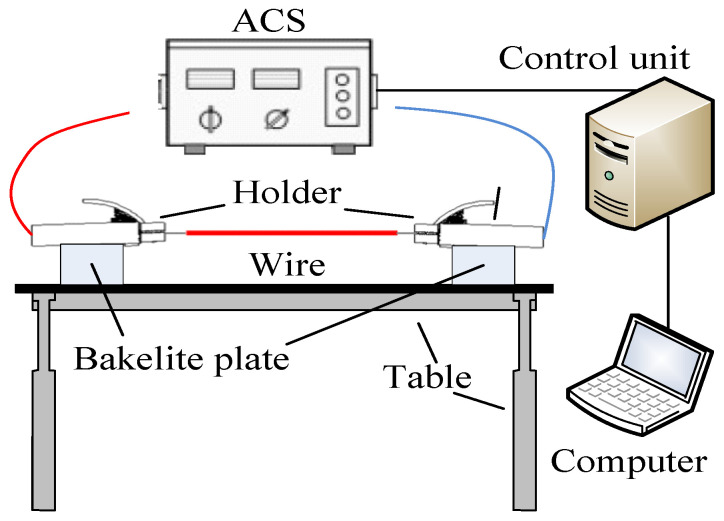
Schematic of the experimental apparatus.

**Figure 2:**
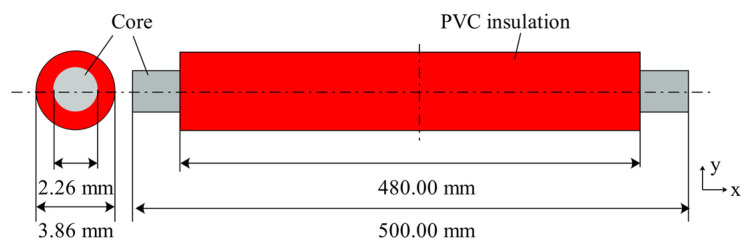
Wire configuration (mm).

The experimental device comprises an alternating current source (ACS) and a control unit, as shown in Figure 1. The experimental wires included AA8176 aluminum alloy wires and Al wires commonly used in the industry. The chemical composition of the wires is shown in Table 1, where the data are sourced from the wire manufacturer (Zhongde cable, Shijiazhuang, China). Figure 2 shows a schematic diagram of the experimental wire structure. The diameter of the wire is 4 mm^2^, the rated current is 32 A, each section of wire is 500 mm long, and 10 mm of the insulation layer is stripped off at both ends for connection to the experimental device.

Babrauskas V [10] found that more than 300% overcurrent ignites nearby combustibles and cause electrical fires. In this article, 400%, 500%, 600%, and 700% overcurrent faults are simulated on the basis of previous studies. Based on the limitations of medium- and high-voltage electrical fault simulation experimental conditions and safety factors, the experimental voltage is 220 V, and the current is 128 A, 160 A, 192 A and 224 A. In order to ensure that the experimental results are more accurate and reduce errors, five sets of parallel experiments are carried out for each experimental condition. After an overcurrent fault occurs in the wires, they fuse under the action of Joule heat. As the temperature rises, electrons on the surface of the molten pool are accelerated from the cathode to the anode and collide with the metal vapor to generate a metal arc, which leads to arc beads forming under the action of arc heat and force [27].

For microscopic analysis and metallographic examination, overcurrent arc beads (OABs) were perpendicularly cut from the overcurrent wires, from the fusion point to 10 mm from this point. The metallographic samples were prepared in accordance with ASTM standards [28,29], and the samples were ground with 240-, 800-, and 2000-grit sandpapers and polished with flannelette. An HF acid solution (concentration H_2_O:HF = 5%:95%) was used to corrode the sample until the metallographic structure appeared, and this process was repeated as often as necessary. The same was true for samples prepared at the rated current.

Metallographic analysis was carried out in the areas of OABs used Axio Vert. A1 metallographic microscope (Zetiss, Oberkochen, Germany), and the Image-Pro-Plus software (Media Cybernetics, Rockville, MD, USA) was used to calculate the grain diameters.

Microscopic examination of cross-sections of OABs was performed on a QUANTA FEG 450 field emission scanning electron microscope (Thermo Fisher Scientific, Waltham, MA, USA). Composition measurements of OABs were performed with an Ultim Max X-ray spectrometer (Oxford Instruments, Abingdon, UK)

Thermogravimetric and differential thermal analyses of wires were performed using a STA-449-F3 thermogravimetry-differential scanning calorimeter ( NETZSCH, Selb, Germany), and the temperature was increased from 30 °C to 1000 °C at a rate of 10 K/min.

## 3. Results and Discussion

### 3.1. Properties of Wires at the Rated Current

**Figure 3:**
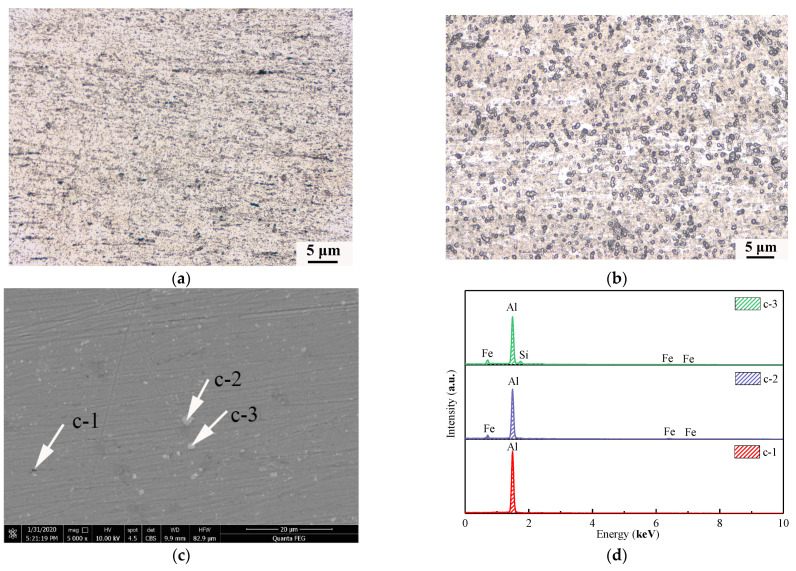
Macrostructural characteristics of wires at their rated currents: (**a**) metallographic structure of aluminum wires and; (**b**) aluminum alloy wires; (**c**); SEM image; (**d**) EDS analysis.

Figure 3 shows the microscopic characteristics of wires under different conditions. Figure 3a is the metallographic structure of the aluminum wires at its rated current; the metallographic structure is elongated along the deformation direction with oriented fibrous features. Figure 3b is the metallographic structure of the aluminum alloy wires at its rated current; the metallographic structure of the aluminum alloy wires is different from that of the aluminum wires at its rated current. For the aluminum alloy wires at its rated current, no crystal grains and grain boundary characteristics are found in the metallographic structure. The α-Al matrix is uniformly distributed with smaller particle sizes and larger numbers of irregular spherical particles. SEM/EDS is used to explore the spherical particles in the metallographic structure, as shown in Figure 3c,d. Parts c-1, c-2 and c-3 in Figure 3c were scanned. According to the results of Figure 3d, the black point c-1 is the α-Al matrix. The second phase in the aluminum alloy wires could be the result of removal of material by grinding and polishing, or it may be a pit formed by the larger particle size of the polishing liquid during polishing. According to the results of Figure 3d, points c-2 and c-3 are Al-Si and Al-Fe binary phases, respectively.

**Figure 4:**
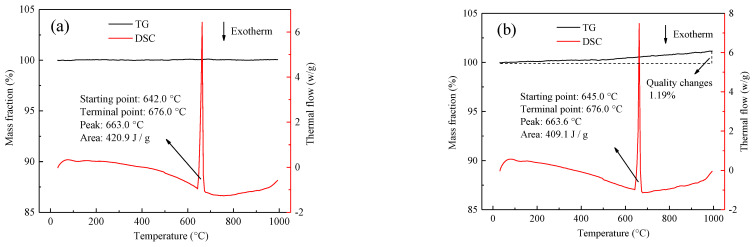
TG/DSC curve of wires in an air atmosphere: (**a**) aluminum alloy wires; (**b**) aluminum wires.

The metallographic structures of aluminum wires and aluminum alloy wires at their rated currents are different, whether the binary phase particles in aluminum alloy wires affect the thermophysical properties of the wires. The TG/DSC curves of aluminum alloy and aluminum wires are shown in Figure 4, where Figure 4a shows the TG/DSC curve of aluminum alloy wires. The aluminum alloy wires were heated to 1000 °C in an air environment, and the quality of the aluminum alloy wires was determined. The loss was almost 0%, and the DSC curve shows an endothermic peak at 663.0 °C. According to Figure 4b, which shows the TG/DSC curve of aluminum wires, the DSC curve between 652.1 °C and 676.0 °C exhibits the same endothermic peak as that of the aluminum alloy wires. The difference is that the aluminum wires gain weight due to oxidation. The increase is 1.19%, but the DSC curve of the aluminum wires does not show an exothermic oxidation peak. The exothermic oxidation peak is speculated to be small and covered by the endothermic peak. The binary phase in the aluminum alloy wires does not change the thermophysical properties of the aluminum wires, but it improves the oxidation resistance of the aluminum alloy and gives the aluminum alloy wires good mechanical properties [30,31].

### 3.2. Metallographic Structure of Aluminum Alloy OABs

**Figure 5:**
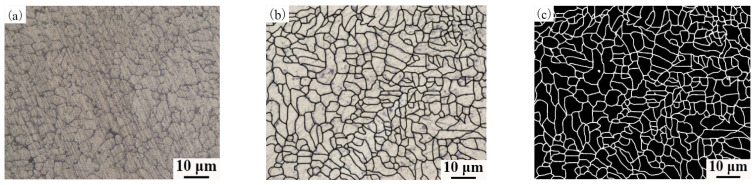
Metallographic structure processing for OABs: (**a**) primary metallographic structure; (**b**) after the grains were resolved more sharply; (**c**) final metallographic structure.

To scientifically and quantitatively analyze the grain diameter, the metallographic structure needs to be processed. Figure 5 shows the binarization process for the metallographic structure of the aluminum alloy wires OABs. Figure 5a shows the original metallographic structure of the aluminum alloy wires. First, the Photoshop software was used to sharpen the appearance of the grains, as displayed in Figure 5b. Second, the Image-Pro-Plus software was used to process the image, the pixels with a gray level of 0 were defined as grain boundaries, and the pixels with a gray level of 255 were defined as grains. In addition, the Count/Size option was used and the Automatic Bright Objects feature was selected to evaluate the grain size.

**Figure 6:**
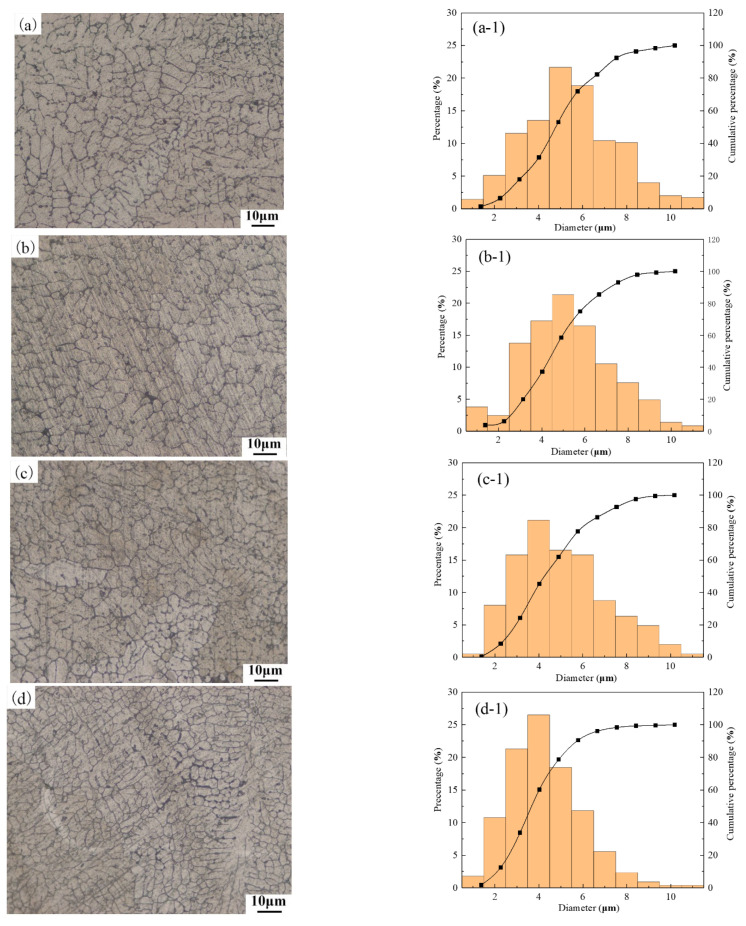
Microstructure and grain size distribution of OABs: (**a**) and (**a-1**) 128 A; (**b**) and (**b-1**) 160 A; (**c**) and (**c-1**) 192 A; (**d**) and (**d-1**) 224 A.

Figure 6 shows the metallographic structure and the corresponding particle size distribution of the OABs for different currents. Figure 6a–d shows that the second phase is not found in the OABs, the grain boundaries are well-defined, and the crystal grains have a dendritic cellular structure. When the current is increased from 128 A to 224 A, the metallographic structure of the OABs remains unchanged. The distribution of the OAB grains is relatively narrow, mainly concentrated between 3 μm and 6 μm.

Compared with the metallographic structure characteristics at the rated current, the second phase is integrated into the matrix after the overcurrent fault occurs in the wires. According to the arc theory, the following formula can be obtained [32]:

(1) $$T_{\mathrm{arc}}=6500\;\;\;I_{a}\leq 4.5A$$

(2) $$T_{\mathrm{arc}}=4010+1658\mathrm{In}I_{a}\;\;\;I_{a}>4.5A$$

In the formula, $T_{\mathrm{arc}}$ is the arc temperature and $I_{a}$ is the current. Formulas (1) and (2) show that the theoretical arc temperature is at least 6500 K. When the current increases, the arc temperature gradually rises, which can be understood from the principle of metal solidification. The higher temperature results in an easier diffusion of the second phase. Therefore, a large amount of the second phase is dissolved into the matrix.

**Figure 7:**
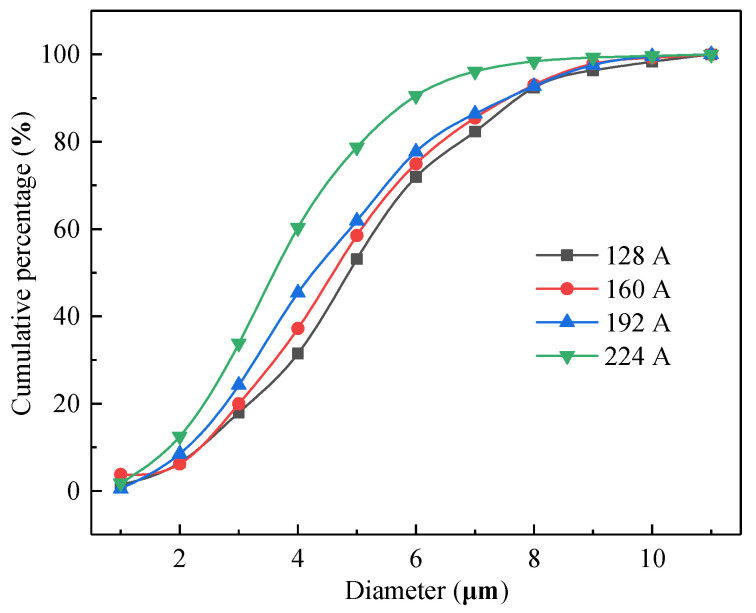
Cumulative percentage of grain size at different currents.

**Figure 8:**
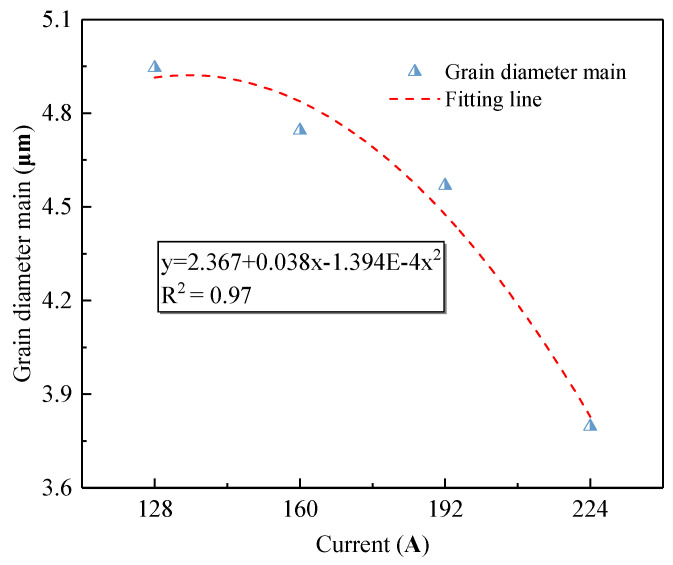
Mean grain diameter of OABs for different overcurrents.

Figure 7 shows the cumulative percentage of grain sizes at different currents. Figure 7 shows that the larger the current is, the steeper the slope of the cumulative curve, especially when the grain size is smaller. The relationship between the average particle size of the OABs and the current is shown in Figure 8. According to Figure 8, as the current increases, the average grain size of the crystals gradually decreases. Therefore, the current is concluded to have a refining effect on the OAB grains. Nonlinear fitting of the grain size and current was carried out, and the overcurrent intensity and the grain size satisfied a quadratic function. The change in grain size is related to the degree of undercooling. At 128 A, the Joule heat and arc temperature are lowest, the undercooling degree of the molten marks is smaller, the radius of the crystal nuclei is larger, and the final grain size is largest. Conversely, at 224 A, the Joule heat and arc temperature are highest, the degree of undercooling is lower, the nucleation radius is smallest, and the grain size is relatively small.

### 3.3. Influence of Alloying Elements on the Microscopic Morphology of OABs

**Figure 9:**
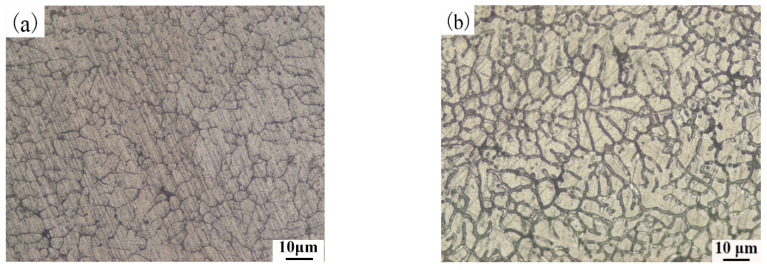
Metallographic structure for OABs with a 160 A overcurrent: (**a**) aluminum alloy wires; (**b**) aluminum wires.

Figure 9 shows the metallographic structures of the 160 A OABs in aluminum alloy and aluminum wires. The aluminum alloy and aluminum wires OABs have the same structure, consisting of cellular dendrites, and the grain boundaries are well-defined and complete. Notably, the aluminum alloy wire OABs are finer, whereby the alloying element refined the grains.

**Figure 10:**
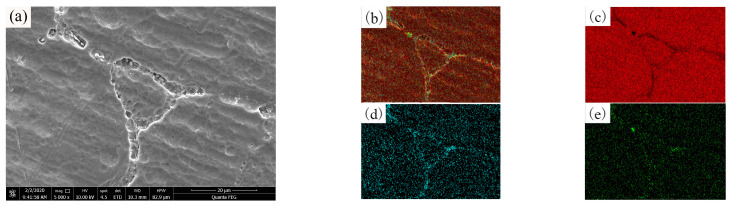
SEM/EDS images for OABs of aluminum alloy wires with a 160 A overcurrent: (**a**) SEM image; (**b**) total element mapping; (**c**) Al mapping; (**d**) Fe mapping; (**e**) Si mapping.

The second phase dissolves and diffuses after an overcurrent fault occurs in the aluminum alloy wires. SEM/EDS was used to map and analyze the OABs of the aluminum alloy wires and aluminum wires. Figure 10 shows the microscopic morphology and element distribution of the OABs of aluminum alloy wires with a 160 A overcurrent. Figure 10b shows that the distribution of elements in the grains is not uniform; as shown in Figure 10d, Fe segregates at the grain boundaries. Because of Joule and arc heating, the molten marks cause the second phase to dissolve and diffuse. The atomic radius of Fe is larger than that of Al. If an Fe atom enters the lattice, it can cause large lattice distortions, which increase the energy of the system. However, the grain boundary atoms are arranged more loosely, and the distortion energy caused by Fe in the grain boundaries is much smaller than that generated by Fe in the lattice. To reduce the energy of the system, the Fe atoms can only be arranged at irregular grain boundaries, and thus, grain boundary segregation occurs. An undissolved Si-rich phase is also found in Figure 10e. Because the arc temperature distribution is not uniform, a part of the Al-Si phase is not melted.

**Figure 11:**
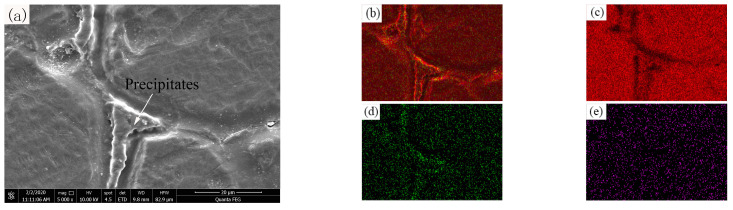
SEM/EDS images for OABs of aluminum wires with a 160 A overcurrent: (**a**) SEM image; (**b**) total element mapping; (**c**) Al mapping; (**d**) O mapping; (**e**) Cl mapping.

Figure 9 shows that the metallographic structure of the aluminum wires OABs has coarse grain boundaries. Figure 11 presents the microscopic morphology and mapping of the OABs of the aluminum wires with a 160 A overcurrent. Figure 11a shows that the aluminum wires OABs have precipitate phases at the grain boundaries. Figure 11b,c shows that the precipitate phases at the grain boundaries are α-Al, and the distribution of elements between the grains and the grain boundaries of the aluminum wires is uniform. Since the grain boundary energy is lower, the solute aggregates at the grain boundaries. Furthermore, the undercooling of the OABs is large, causing the final solidified grain boundary solute to fail to diffuse in time. Interestingly, O was found in the OABs of the aluminum wires because the aluminum wires was oxidized in the molten state. The TG/DSC data shown in Figure 4b support this conclusion. In addition, Cl was also found in the OABs, which is due to the entrainment of combustion products from the insulation when in the molten state [26].

## 4. Conclusions

This paper analyzes the effects of current on the microscopic characteristics of aluminum alloy wire OABs and clarifies the difference between OABs in aluminum alloy and aluminum wires. The conclusions are as follows:

(1) Under the rated current of aluminum alloy wires and aluminum wires, there are no obvious grain boundaries or grain characteristics in the metallographic structure of the wires. Furthermore, large amounts of Al-Si and Al-Fe binary phases are distributed in the α-Al matrix.

(2) After an overcurrent occurs in the wires, the metallographic structure of the aluminum alloy wire OABs consists of cellular dendrites, and the second phase disappears. As the current increases, the grain size gradually decreases.

(3) The microstructure characteristics of the OABs of aluminum alloy wires are the same as those of aluminum wires. Nevertheless, the second phase in the OABs of aluminum alloy wires has a refining effect on the grain boundaries, and segregation occurs at the grain boundaries. The OABs of aluminum alloy wires have finer grain boundaries than those in aluminum wires, and there is a precipitated phase of α-Al in the OABs of aluminum wires.

## Figures and Tables

**Table 1:** Chemical compositions of wires (mass fraction/%).

Sample	Chemical Compositions (Mass Fraction/%)								
	Si	Fe	Cu	Mg	Zn	B	Else		Al
							Single	Total	
AA8176	0.03–0.15	0.40–1.0	-	-	0.10	-	0.05	0.15	Balance
Al	-	-	-	-	-	-	-	-	100

## Data Availability

The data presented in this study are available on request from the corresponding author.

## References

[1] Liu D.Y., Li W.J., Han Y., Gao Q., Ma G. (2014). Alloying design of a thermal-resistant aluminum alloy conductor material with high conductivity. Trans. Mater. Heat Treat..

[2] Sambor W., Miroslaw O., Lukasz W. (2015). Development of innovative aluminum alloys for production of overhead electrical conductors. Light Met. Age.

[3] Han X., Li Y., Pang H., Li C., Cui L., Wang J., Liu Y. (2020). Effect of isothermal annealing on microstructure and properties of 8030 aluminum alloy wire. Heat Treat. Metals..

[4] Tremblay K.J. (2015). Poor electrical connection ignites church fire. Epigenomics.

[5] Hong X.Y. (2014). The Comparative research of melted trace due to fire-burning and short-circuit. J. Liaoning Univ..

[6] Babrauskas V. (2003). Ignition Handbook.

[7] Babrauskas V. (2006). Mechanisms and Modes for Ignition of Low-voltage, PVC-insulated Elec-trotechnical Products. Fire Mater..

[8] Xiao K.C., Xue Y.X., Yang L., Wei F.W., Lei B., Liu B.W., Bo W. (2020). Investigation of evolution process and molten marks characteristics of overcurrent fault. J. Xian Univ. Sci. Technol..

[9] Iwashita T., Hagimoto Y., Sugawa O. (2017). Characterization of arc beads on energized conductors exposed to radiant heat. Fire Mater..

[10] Babrauskas V. (2008). Research on electrical fires: The state of the art. Fire Saf. Sci..

[11] Babrauskas V. Arc Mapping: A Critical Review. Fire Technol..

[12] NFPA921 (2017). Guide for Fire and Explosion Investigations.

[13] Erlandsson R., Strand G. (1985). An investigation of physical characteristics indicating primary or secondary electrical damage. Fire Saf. J..

[14] Takaki A. (1971). On the Effect of Thermal Histories upon the Metallographic Structure of Electric Wires.

[15] Babrauskas V. (2004). Arc Beads from Fires: Can ‘Cause’ Beads be Distinguished from ‘Victim’ Beads by Physical or Chemical Testing?. J. Fire Prot. Eng..

[16] Wei G., Ying W., Shujun L., Liantie W. (2010). Study on the in-depth composition of beads formed by fuse breaking of electric wire at different oxygen concentrations by auger electron spectroscopy. Spectrosc. Spectr. Anal..

[17] Ying W., Qing S.M., Xin M.W., Wei G., Man D. (2010). XPS Analysis of beads formed by fuse breaking of electric copper wire. Spectrosc. Spectr. Anal..

[18] Hai R.W., Jian Y.L., Hao W.Y., Dong L. (2012). The Microstructure and phase composition of metal melting marks caused by different fire. Spectrosc. Spectr. Anal..

[19] Park J., Kang J.H., Lee E.P., Young H.K., Sun B.B. (2021). New approach to distinguish copper molten marks based on quantitative microstructure analysis using electron backscatter diffraction. Fire Technol..

[20] Kuan H.L., Yung H.S., Guo J.C., Jaw M.C. (2015). Microstructural study on molten marks of fire-causing copper wires. Materials.

[21] Chen C.Y., Ling Y.C., Wang J.T., Chen H. (2003). SIMS depth profiling analysis of electrical arc residues in fire investigation. Appl. Surf. Sci..

[22] Dongbai X., Wen W., Shilei L., Shi D. (2018). Visual and oxide analysis for identification of electrical fire scene. Forensic Sci. Int..

[23] Chungseog C., Hyangkon K., Kilmok S.A. (2004). Study on the short-circuit characteristics of vinyl cords damaged by external flame. Fire Sci. Eng..

[24] Babrauskas V. (2001). How do electrical wiring faults lead to structure ignitions?. Fire Mater..

[25] Xin J., Huang C.F. (2014). Fire risk assessment of residential buildings based on fire statistics from China. Fire Technol..

[26] Zhi J.Y., Shuang S.C., Jun D., Xue X.X., Wei F.W. (2020). Microstructural Characteristics of Arc Beads with Overcurrent Fault in the Fire Scene. Materials.

[27] Xue X.X. (2020). Investigation of the Evolution Process and Microstructure of Molten Marks after Overcurrent Fault of Aluminum Alloy Wire.

[28] ASTM E3-11 (2017). Standard Practice for Preparation of Metallographic Specimens.

[29] ASTM E407-07 (2015). Standard Practice for Macroetching Metals and Alloys.

[30] Feng M., Guang Y.Y., Zhen J.X., Zhi Q.C., Tong M.W. (2015). Effect of Eu addition on the microstructures and mechanical properties of A356 aluminum alloys. J. Alloy. Compd..

[31] Fang H., Sun J., Wang H., Deng Y. (2016). Influence of alloy added trace cerium on microstructure and properties of 7136 aluminum. J. Chin. Soc. Rare Earths.

[32] Hurley M.J., Gottuk D.T., Kuligowski E.D., Puchovsky M., Torero J.L., Watts J.M., Wiczorek C.J. (2016). SFPE Handbook of Fire Protection Engineering, Society of Fire Protection Engineers.

